# The Role of the Sleep Centre in the Future of Sleep Medicine. How Should It Be Organised? Will There Still Be Sleep Inpatients in the Future?

**DOI:** 10.1111/jsr.70092

**Published:** 2025-05-15

**Authors:** Thomas Penzel, Matthew Salanitro, Ingo Fietze

**Affiliations:** ^1^ Interdisciplinary Sleep Medicine Center, Charitecenter for Pneumology Charite Universitätsmedizin Berlin Berlin Germany

**Keywords:** home sleep testing, polysomnography, sleep centre, wearables

## Abstract

Sleep medicine centres play a pivotal role in diagnosing, treating and researching sleep disorders, with structures that range from large, university‐based institutions to smaller, community‐led clinics. These centres operate at the intersection of clinical care and academic innovation, combining personalised diagnostics and therapy with the evaluation and integration of emerging technologies. As the demand for sleep‐related healthcare continues to grow, sleep centres must evolve to accommodate both increasing patient loads and rapid technological advancements. Outpatient departments serve as critical entry points for patients, enabling structured assessments through consultations, questionnaires and objective tools such as actigraphy and home polygraphy. These are increasingly complemented by digital solutions, including telemedicine, automated sleep diaries and remote prescription management. Such tools improve accessibility, streamline workflows and enhance continuity of care. Despite technological progress, sleep laboratories remain indispensable for diagnosing complex disorders like sleep apnea and hypersomnias, where in‐lab polysomnography and real‐time therapy adjustments are essential. However, advances in portable and wearable devices are creating viable home‐based alternatives for selected cases, contributing to reduced lab wait times and broader patient reach. Research and education are foundational to advancing the field. Ongoing updates to sleep medicine curricula, alongside rigorous clinical studies on novel diagnostics and AI tools, ensure that care remains evidence‐based and future‐ready. Sleep centres, as multidisciplinary hubs, will continue to merge clinical practice, innovation and training—shaping a future where sleep healthcare is more personalised, data‐driven and accessible without compromising quality or equity. Together, these developments signal a transformative era in sleep medicine, where integrated, technology‐enhanced care will redefine how we understand, diagnose and treat sleep disorders.

## 1 Introduction

Sleep medicine centres are firmly embedded in the healthcare systems of many countries, operating both in university hospitals and larger community hospitals. Most offer the full spectrum of diagnostic and therapeutic services for recognised sleep disorders; however, some centres concentrate on only a subset of conditions. These dedicated units are typically limited to university hospitals or to community hospitals led by clinicians with a strong personal commitment to the field and their scope and success often hinge on the expertise and enthusiasm of a single sleep physician.

Regardless of size or setting, every centre fulfils a multifaceted role. Clinically, it delivers comprehensive diagnostics, personalised treatment plans and patient education to improve sleep‐health outcomes (Hartley et al. 2024). Academically, it drives research, evaluates cutting‐edge technologies and collaborates across disciplines to advance our understanding of sleep. In this way, sleep medicine centres not only meet today's patient needs but also help shape the future of sleep disorder management.

As we progress further into the digital age, sleep centres have the opportunity to embrace emerging technologies that enhance both patient care and operational efficiency. From portable sleep trackers to AI‐powered applications optimising administrative and clinical tasks, these innovations should be seamlessly integrated into practice (Perez‐Pozuelo et al. 2020)—not as additional burdens for patients or staff, but as tools that alleviate workload and improve accessibility to manage sleep problems and sleep disorders.

## 2 Outpatient Department

For most patients, the journey begins at the sleep centre's outpatient department, where they attend an initial consultation with a physician to assess potential sleep disorders. In addition to this consultation, patients complete preliminary questionnaires tailored to their specific sleep complaints, helping physicians refine their diagnostic approach. When further evaluation is necessary, patients undergo objective preliminary measurements suited to their condition—such as actigraphy for insomnia and circadian rhythm disorders, or polygraphy (PG, commonly known as a home sleep apnea test, typically involving four or six channels and formerly referred to as level‐3 devices) for suspected sleep apnea (Ancoli‐Israel et al. 2003; McNicholas 2008).

Based on these findings, individualised treatment pathways are developed. These may include admission to the sleep lab for further outpatient sleep testing. If possible, treatment recommendations for optimal sleep hygiene, cognitive behavioral therapy, pharmacological or other interventions can be initiated by outpatient management when appropriate. Patients may return for symptom follow‐up, treatment follow‐up or for regular medication prescriptions as part of their continuing treatment.

**FIGURE 1:**
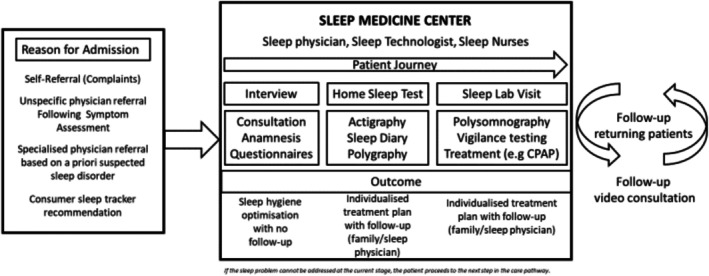
The components for a sleep medicine centre in the future. Many components are already in place today. Some started during the pandemic like video consultations. The main change in future will be a shift in numbers along the arrows indicated. Quantitative assessments are not available until now.

Currently, this process represents the standard of care. However, with technological advancements, we may soon see a shift in how sleep centres operate. Initial questionnaires could be completed online before the first consultation, as a first step, reducing administrative delays at the day of the consultation. Medication prescriptions for continuing treatment could be managed via digital apps and sent directly to local pharmacies. Telemedicine in terms of video consultation could expand access to care, enabling remote consultations for patients who live at a greater distance or have difficulties travelling (Ukoumunne et al. 2017; Ng et al. 2025; Shamim‐Uzzaman et al. 2021). All components of organisation are depicted in Figure 1.

Building on these advancements, additional innovations could further improve the efficiency and accuracy of sleep disorder diagnostics and treatment. For example, automated sleep diary apps would enable patients to digitally track their sleep patterns, minimising reliance on subjective self‐reports while offering clinicians more reliable, long‐term data (Schmitz et al. 2022). AI‐assisted diagnostics could analyse actigraphy and PG recordings, enabling physicians to identify sleep disorders more efficiently and optimise treatment decisions by leveraging large databases of previous recordings (Tilmanne et al. 2009). Furthermore, remote home sleep monitoring with portable devices could provide an alternative to in‐lab studies for carefully selected patients, making sleep assessments more accessible without compromising diagnostic accuracy. By thoughtfully integrating these digital solutions, sleep centres can improve resource efficiency while ensuring high‐quality, patient‐centred and individualised care.

It is important to note that while ambulatory home‐based techniques are gaining traction, especially for preliminary assessments and monitoring, most new medications and therapies for sleep disorders are still being tested within controlled sleep laboratory environments. Importantly, there remains a lack of established standard operating procedures for conducting and evaluating these measurements in clinical research, highlighting a critical gap that must be addressed to ensure consistency and reliability if these solutions are to be integrated into standard care.

## 3 Sleep Lab—Patients—Healthcare Staff

Sleep labs are primarily used by patients with sleep‐related breathing disorders, hypersomnias and parasomnias, as these conditions still require in‐lab diagnostic tools like overnight polysomnography (PSG), morning wakefulness tests and multiple sleep latency tests to ensure an accurate, reliable diagnosis (Markun and Sampat 2020). Some conditions also require that treatment be initiated during the lab stay. For instance, patients with sleep apnea often undergo continuous positive airway pressure (CPAP) titration, spending a night in the lab so clinicians can monitor and adjust treatment in real time (McNicholas 2008). If CPAP is ineffective, alternatives such as bi‐level or auto‐bi‐level ventilation or other therapies, including maxillofacial surgery, may be explored. These scenarios underscore the essential role of sleep labs in developing personalised therapy plans. Additionally, for patients with disabilities or those with significant medical or mobility limitations, the hospital environment—offering full nursing support—is irreplaceable, making in‐lab care a necessity.

**FIGURE 2:**
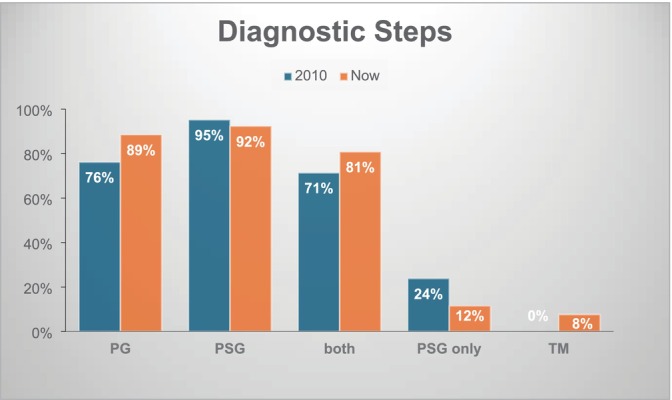
Comparison of the 2010 and 2020 response (percentage of participating countries, 2010: *n* = 21, 2020: *n* = 26) to the survey question on what diagnostic steps were performed. PG, polygraphy; PSG, polysomnography; TM, telemedicine. Figure from (Fietze et al. 2022).

As technology advances, some diagnostic procedures are shifting from strictly in‐lab evaluations to at‐home alternatives. Remote home‐sleep monitoring using compact portable devices is becoming more reliable, reducing the need for in‐lab PSG in carefully selected cases (American Academy of Sleep Medicine 2023a). A Europe‐wide survey by Fietze et al. found that over the past decade, in‐lab PSG appointments for diagnosing and treating sleep disordered breathing have declined, while PG appointments have increased during the same period (Figure 2) (Fietze et al. 2022). However, the broader adoption of PG has been slow due to limited reimbursement in many European countries, restricted access to necessary equipment, and a persistent shortage of trained technologists and sleep physicians capable of supporting outpatient care (Todea et al. 2012).

It is important to note that the use of ambulatory and non‐attended PSG remains insufficiently defined in many European countries. Standard operating procedures for these approaches are lacking, which limits their integration into clinical workflows Fietze et al. (2022). Moreover, the absence of visual observation in non‐attended PSG poses a significant constraint—particularly for diagnosing conditions such as parasomnias and movement disorders during sleep, where visual cues are often essential for accurate assessment.

Automatic analysis represents the next critical step in expanding the use of ambulatory sleep technology. A certified auto‐scoring system for sleep‐stage analysis in PSG is already available (American Academy of Sleep Medicine 2023b), and these state‐of‐the‐art AI models are now capable of identifying sleep stages with accuracy levels comparable to human inter‐scorer agreement, which typically ranges between 82% and 89% (Lee et al. 2022). This ceiling in accuracy is largely due to the fact that AI systems are trained on manual annotations, which themselves contain minor inconsistencies between scorers. As a result, these models cannot yet fully replace human scorers. Nevertheless, when used as assistive tools, AI‐based scoring solutions can significantly accelerate clinical workflows without compromising diagnostic reliability. Previous studies also show that AI performance continues to improve (Moeller et al. 2025). What remains is the development and certification of algorithms for respiratory event scoring and a broader range of ambulatory monitoring techniques. Once these are in place, they could transform clinical routines by automating repetitive tasks and allowing healthcare professionals to focus more directly on patient care.

This wave of technological innovation has the potential to reduce wait times for sleep‐lab admissions by triaging patients more effectively—ensuring that those who truly require in‐lab treatment can access it sooner. A wide array of devices is currently under evaluation as potential surrogates for detecting respiratory events (Burian et al. 2024), which could ultimately aid in diagnosing sleep apnea—the most common sleep disorder still requiring PSG. These include rings, watches, smartphone‐based tests, chest‐worn recorders, wireless skin patches, peripheral arterial tonometry tools, pulse oximeters with built‐in microphone channels, and fully contact‐free sensing technologies (Lyne et al. 2023; Zhao et al. 2022; Chee et al. 2025; Goldstein et al. 2025).

In parallel, similarly promising tools are emerging for insomnia assessment. These include actigraphy, electronic armbands (Grau et al. 2022), smartphone apps and a wide range of wearable technologies—watches, rings (Herberger et al. 2025), smart earbuds, lob‐patch systems, headbands, on‐scalp printed EEG e‐tattoos (Vasconcelos et al. 2025) and even sensors embedded directly into everyday clothing such as T‐shirts (Patterson et al. 2023; de Scalco Vasconcelos et al. 2024; Kim et al. 2023). Together, these innovations offer the potential to decentralise sleep diagnostics and better tailor assessments to real‐world settings.

## 4 Sleep Lab—Epidemiology—Sleep Health

Medical training and education in sleep medicine is a crucial priority for the future of the field. To support this, various educational tracks have been developed, and to feed these programmes with relevant content, a comprehensive, content‐driven curriculum is required. A catalogue of knowledge and skills for sleep medicine was created to guide this curriculum. In its first edition, the catalogue served as the foundation for the examination to become a sleep expert in Europe (Penzel et al. 2014). It was developed through a Delphi round among sleep centres across Europe to ensure it aligned with the needs of clinical practice. This collaborative approach helped ensure the catalogue's relevance and practicality, ultimately serving as a blueprint for education and training.

The catalogue was finalised in 2012 and published alongside the development of an ESRS textbook on sleep research and sleep medicine, with the catalogue reflecting the textbook's table of contents. Over time, new guidelines and technologies, such as telemedicine in sleep medicine, emerged, influencing the field. As sleep disorders gained recognition across various medical specialties—such as neurology and cardiology—the need for tailored sleep medicine education in these fields became evident. This shift required an update to both the ESRS textbook and the catalogue.

The revised version of the catalogue, published in 2021 (Penzel et al. 2021), was designed to reflect these new expectations while still serving as a foundation for examinations to become a European Sleep Expert. The updated catalogue was aligned with the current ESRS textbook, and it continues to underpin the education and certification process for sleep specialists (McNicholas et al. 2025).

As sleep medicine evolves, so too must the methodology of medical education. With new guidelines, updated terminology (e.g., the inclusion of sleep disorders in ICD‐11), and the increasing importance of telemedicine, the next update to the catalogue (Version 3) is currently under development. This revision will focus on clearly defining learning objectives, reflecting current terminology and integrating modern teaching methods to keep pace with the rapidly changing landscape of sleep medicine.

Together, these ongoing updates will shape the future of sleep medicine education, ensuring it remains relevant, timely and aligned with current and emerging practices.

Epidemiological research is becoming increasingly important, as its findings can help shape effective prevention strategies. However, the potential role of restorative sleep in prevention has not been sufficiently explored. Only recently, the recognition of the need for sleep‐inclusive epidemiological studies is growing. Several large‐scale studies have incorporated sleep assessments, including the Sleep Heart Health Study in the United States, the Episono study in Brazil and the SHIP and NaKo studies in Germany. Additionally, the UK Biobank has leveraged actigraphy, a tool primarily designed for analysing activity and movement, to include sleep assessments in its research (Doherty et al. 2017).

At the same time, emerging technologies—particularly consumer wearables like smartwatches and smartphones—are enabling the collection of vast amounts of sleep‐related data across entire populations, as demonstrated in recent global analyses. These datasets hold tremendous potential for advancing sleep epidemiology and developing preventative strategies. However, there is currently no consensus on standards for data collection, analysis or evaluation, which poses a significant challenge to ensuring data quality and comparability across studies.

## 5 Sleep Research Centre

Stepping away from patient care, we now turn to the important work being done at the research department within the sleep centres. This department, typically situated alongside the outpatient departments or sleep labs, is dedicated to advancing the field through research on emerging technologies for diagnosis, treatment and more. Researchers at these centres design, test and report on studies that could revolutionise how sleep disorders are diagnosed and managed (Lenze et al. 2025a, 2025b).

Access to patients is essential for these research centres to continue pushing the boundaries of sleep medicine, as there is still much to learn about the complexities of sleep disorders. A key aspect of research is evaluating new technologies to determine their potential for integration into routine clinical practice. This includes examining innovations such as wearable devices, non‐pharmacological treatments, and AI for sleep scoring and sleep disorder diagnosis—all of which hold the promise of reducing the burden on both healthcare staff and patients. By testing these innovations, research centres play a vital role in assessing whether they can be successfully incorporated into standard care practices, ultimately improving both diagnostic efficiency and treatment outcomes.

## 6 Expertise

As home sleep tests begin to replace more PSG investigations, their application will become more practical outside of traditional sleep medicine. However, this shift will also require expertise from the users of these tests. This presents a future challenge that includes expanding robust networks, enhancing training and continuing education and establishing standards and quality controls for different testing systems (Lenze et al. 2025a). It is important to remember that a sleep study alone does not make a diagnosis. Instead, it is the combination of measurements, subjective complaints, anthropometric data and comorbidities that lead to an accurate diagnosis. One of the future challenges for AI will be integrating all these diverse data points effectively.

## 7 Conclusion

As sleep medicine continues to evolve, the organisation of sleep centres must adapt to changing patient needs and technological advancements. There is a clear shift towards more outpatient care, driven by the increasing volume of patients and the growing demand for sleep optimisation. However, inpatient care remains essential for certain complex assessments—as well as confirming diagnoses, clarifying pathophysiology in difficult cases and validating new technologies. For instance, procedures like CPAP titration for sleep apnea require a controlled sleep lab environment, where patient responses can be closely monitored, something that home monitoring technologies cannot replicate.

At the same time, outpatient care must integrate new technologies, such as consumer sleep trackers and wearable devices, into daily practice. This will help address the rising number of patients with sleep disorders while also meeting the demands for health optimisation. As these technologies evolve, it is critical to network all components of sleep medicine, ensuring seamless integration of data from home sleep recordings, PSG, treatment monitoring (e.g., CPAP adherence) and follow‐up studies. However, such integration must be carefully managed, prioritising privacy and data security.

Although home‐based monitoring and AI‐assisted diagnostics show great promise, they cannot fully replace the personalised care provided by in‐person visits. There is a fine balance between leveraging the convenience of remote care and maintaining the precision and individualised treatment that in‐lab assessments offer. The future of sleep medicine will likely see ambulatory diagnostics playing a larger role, but inpatient care and PSG will continue to be integral to ensuring accurate diagnoses and effective treatments.

## Author Contributions

**Thomas Penzel:** conceptualization, methodology, data curation, supervision, writing – original draft, writing – review and editing, investigation. **Matthew Salanitro:** writing – review and editing, investigation, validation. **Ingo Fietze:** writing – review and editing, investigation.

## Conflicts of Interest

The authors declare no conflicts of interest.

## Data Availability

Data sharing not applicable to this article as no datasets were generated or analysed during the current study.
